# Optimized protocol for tRNA identification in the ribosomal complexes from human cell lines

**DOI:** 10.1016/j.xpro.2021.100615

**Published:** 2021-06-17

**Authors:** Tsuyoshi Udagawa, Moeka Seki, Toshifumi Inada

**Affiliations:** 1 Graduate School of Pharmaceutical Sciences, Tohoku University, Sendai, Miyagi 981-8567, Japan; 2 The institute of Medical Sciences, The University of Tokyo, Minato-ku, Tokyo 108-8639, Japan

**Keywords:** Cell separation/fractionation, Molecular Biology

## Abstract

Here, we describe a protocol for tRNA identification in the 60S ribosome-nascent peptide complex co-purified with Nuclear Export Mediator Factor (NEMF), a responsible factor for C-terminal alanine and threonine tailing of the nascent peptide. Our protocol is based on regular reverse transcription followed by quantitative Polymerase chain reaction (PCR). Although this method cannot distinguish between amino acid-charged and uncharged and base-modified and unmodified tRNAs, it is a convenient way to estimate the relative level of tRNA species and thus can be useful for researchers.

For complete details on the use and execution of this protocol, please refer to Udagawa et al. (2021).

## Highlights

• Strategy to obtain the ribosomal complex from mammalian cells
• Purification of the ribosomal complex with sucrose density gradient centrifugation
• A simple protocol to quantify the levels of individual tRNA species by RT-qPCR

## Graphical abstract

**Figure:**
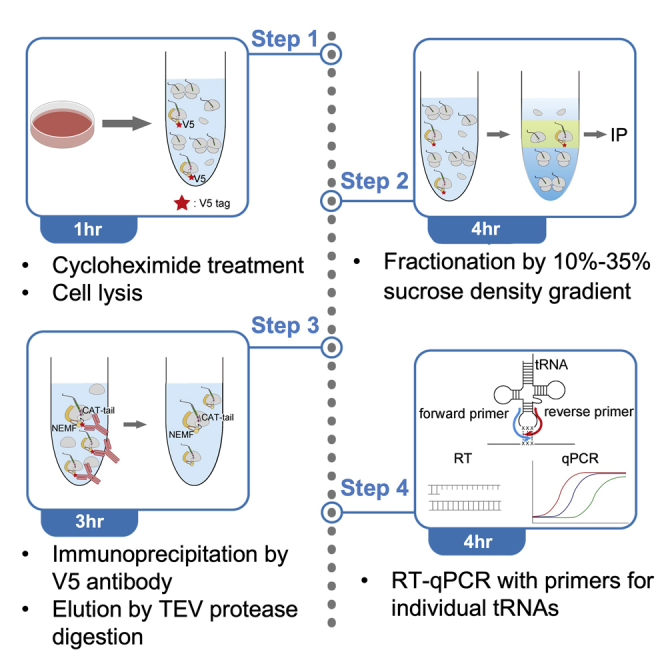

## Teaser

Here, we describe a protocol for tRNA identification in the 60S ribosome-nascent peptide complex co-purified with NEMF, a responsible factor for C-terminal alanine and threonine tailing of the nascent peptide. Our protocol is based on regular reverse transcription followed by quantitative PCR. Although this method cannot distinguish between amino acid-charged and uncharged and base-modified and unmodified tRNAs, it is a convenient way to estimate the relative level of tRNA species and thus can be useful for researchers.

## Before you begin

**Figure 1:**
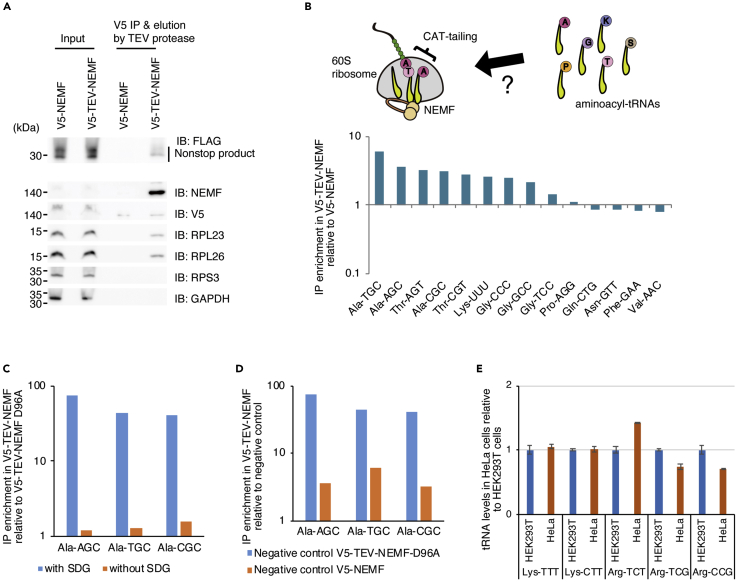
Purification of NEMF-60S RNC and the detection of tRNAs in NEMF-60S RNC (A) Purified NEMF-60S RNC was analyzed by western blotting using the antibodies indicated on the right. V5-NEMF without a TEV protease recognition motif was used as a negative control. Nonstop product and large ribosomal proteins were co-purified with V5-tagged NEMF. Note that the similar data using V5-TEV-NEMF D96A mutant was shown in the original article (Udagawa et al., 2021). (B) Schematic drawing the NEMF-60S RNC and RT-qPCR result of the tRNAs in the NEMF-60S RNC prepared as in A. The immunoprecipitation (IP) enrichment was calculated using Cq values in the V5-NEMF without a TEV protease recognition motif as a negative control. Note that the similar data using V5-TEV-NEMF D96A mutant was shown in the original article (Udagawa et al., 2021). Representative data from 5 independent experiments. (C) The immunoprecipitation (IP) enrichment of alanyl tRNAs in the NEMF-60S RNC prepared with or without sucrose density gradient (SDG) centrifugation step. Representative of 3 independent experiments (D) The IP enrichment of alanyl tRNAs in the NEMF-60S RNC using V5-NEMF or V5-TEV-NEMF D96A mutant as a control. Representative of 3 independent experiments (E) RT-qPCR measurement of both tRNA^Lys^ and tRNA^Arg^ levels in HeLa cells compared to HEK293T cells (n = 3, mean ± s.e.m.).

The protocol described here was used to identify tRNA species in the NEMF- 60S ribosome-nascent peptide complex (NEMF-60S RNC) (Udagawa et al., 2021). For this purpose, relatively large amount of the cells is required for reliable detection of tRNAs by reverse transcription followed by quantitative PCR (RT-qRCR). Cell cultures should be expanded as described below prior to transfecting cells with V5-tagged NEMF. Besides, we include the purification step of 60S ribosomal complexes by sucrose density gradient centrifugation prior to the immunoprecipitation of V5-tagged NEMF, which drastically improves specific detection of individual tRNAs in NEMF-60S RNC (see Figure 1C). Finally, the immunoprecipitated complexes should be eluted from the beads, which reduce nonspecific detection of free tRNAs. We employed TEV protease digestion of the V5-tagged NEMF (V5-TEV-NEMF) which has a TEV protease recognition motif between V5 tag and NEMF. Other tag and elution method such as FLAG tag system may be used alternatively. When this protocol is applied to other experiments such as the identification of tRNAs in the stalled 80S ribosomes or the ribosomes localized to a specific area in the cells, the cell culturing scale, the requirement for sucrose density gradient purification step, and the choice of the tag used for immunoprecipitation should be determined individually.

Expression vectors should be prepared in advance using transfection-grade plasmid DNA purification kit according to the manufacture’s instruction. PEI-Max transfection reagent, D-PBS, protease inhibitor solution, cycloheximide solution, lysis buffer, sucrose buffers, and buffer TEV should be prepared in advance as described below. If needed, protease inhibitors, RNase inhibitor, DTT, β-mercaptoethanol, and/or cycloheximide are supplemented to the buffers immediately before use.

### Preparation of PEI-Max transfection reagent

**Timing: 30 min**

1. Warm up 80 mL of ddH_2_O to about 50°C on a heating magnetic stirrer.
2. Weigh 100 mg of PEI-Max (Polysciences) and pour into the warmed ddH_2_O on a stirrer.
3. Adjust pH to 7.4 with 1N NaOH and fill up to 100 mL with ddH_2_O
4. Filter-sterilize the solution.
5. Separate into 10 mL aliquots in 15 mL tubes.
6. Store at −80°C. Once thawed, the aliquot should be kept at 4°C for up to a year.

### Preparation of protease inhibitor solution

**Timing: 10 min**

7. Dissolve one tablet of complete mini protease inhibitor cocktail (Roche) with 0.5 mL of ddH_2_O in a 1.5 mL microcentrifuge tube
8. Separate into 30 μL aliquots in 1.5 mL microcentrifuge tubes
9. Store at −80°C for up to 2 months and thaw immediately before use. Avoid freeze/thaw cycles.

### Preparation of cell cultures

**Timing: 5 days**

The cell culturing condition described below is for the purification of the ribosomal complexes from two genotypes; V5-TEV-NEMF and the CAT-tailing-deficient mutant (V5-TEV-NEMF D96A) (Udagawa et al., 2021) or the one without a TEV protease recognition motif (V5-NEMF) (see below). The culturing scale should be determined individually depending on the purpose of the experiment.

10. Scaling up the HEK293T cell cultures (10 cm dish).
  a. Wash HEK293T cells on a 10 cm dish at 70% confluency with 3 mL of D-PBS at 20°C–24°C.
  b. Remove the D-PBS and detach the cells with 1 mL of 0.25% trypsin-EDTA at 20°C–24°C for 2 min.
  c. Gently pipet the cells with 9 mL of the medium (DMEM with 10% FBS)
  d. Plate 1.5 mL of the cell suspensions per dish in three 10 cm dishes with 8.5 mL of the medium per dish.
  e. Incubate the cells in a 5% CO_2_ incubator for two days.
11. Scaling up the HEK293T cell cultures (10 cm dish to 15 cm dish).
  a. Prepare the cell suspensions from three 10 cm dishes as above, yielding 30 mL of the cell suspensions.
  b. Plate 4 mL of the cell suspensions per dish in six 15 cm dishes with 21 mL of the medium per dish.
  c. Incubate the cells in a 5% CO_2_ incubator for two days.
12. Scaling up the HEK293T cell cultures (15 cm dish to 500 cm^2^ square dish).
  a. Wash the cells on a 15 cm dish at 70% confluency with 5 mL of D-PBS at 20°C–24°C.
  b. Remove the D-PBS and detach the cells with 2 mL of 0.25% trypsin-EDTA at 20°C–24°C for 2 min.
  c. Gently pipet the cells with 8 mL of the medium.
  d. Repeat for all six 15 cm plates, yielding 60 mL of the cell suspensions.
  e. Plate 15 mL of the cell suspensions per dish in four 500 cm^2^ square dishes with 70 mL of the medium per dish.
  f. Incubate the cells in a 5% CO2 incubator for one day.

***Note:*** You may add antibiotic-antimycotic solution to the medium to avoid contaminations at step 10 and 11. However, at step 12, do not add antibiotic-antimycotic solution to the medium.

## Key resources table

**Table:** 

REAGENT or RESOURCE	SOURCE	IDENTIFIER
**Antibodies**		

Mouse anti-V5 tag, clone SV5-Pk1	Bio-Rad	MCA1360
Mouse anti-FLAG, clone M2	Sigma	F-3165
Rabbit anti-NEMF	Thermo	PA5-36308
Rabbit anti-Rpl23	Abcam	ab112587
Rabbit anti-Rpl26	Bethyl Laboratories	A300-686A
Rabbit anti-Rps3	Bethyl Laboratories	A303-841A
Mouse anti-GAPDH	MBL	M171-3

**Chemicals, peptides, and recombinant proteins**		

DMEM medium	Nacalai	08458-16
FBS	Thermo	26140-079
0.25% Trypsin-EDTA	Nacalai	32777-44
Opti-MEM reduced serum medium	Thermo	31985070
PEI-Max reagent	Polysciences	24765-1
Cycloheximide	Nacalai	06741-04
cOmplete, Mini, EDTA-free Protease Inhibitor Cocktail	Roche	11836170001
RNasin RNase inhibitor	Promega	N2115
D-PBS powder	Nacalai	07269-84
Protein G Dynabeads	Thermo	10003D
AcTEV Protease	Thermo	12575015
HEPES	Wako	340-01376
NaCl	Nacalai	31320-76
MgCl_2_	Wako	136-03995
Igepal CA-630	Sigma	I8896
Sucrose	Nacalai	30406-25
DTT	Nacalai	14128-91
β-Mercaptoethanol	Nacalai	21438-82
Glycerol	Wako	072-04945
Acidic phenol	Wako	315-90291
Chloroform	Wako	038-02606
20 mg/mL Glycogen Solution	Wako	076-06621
3 M Sodium acetate	Ambion	AM9740
Ethanol	Wako	057-00451

**Critical commercial assays**		

ReverTra Ace RT Kit	Toyobo	TRT-101
KAPA SYBR FAST qPCR Mix	NIPPON Genetics	KK4602

**Experimental models: cell lines**		

HEK293T	ATCC	CRL-11268
HeLa	ATCC	CCL-2

**Oligonucleotides**		

Ala-TGC-F: 5′-TGGTAGAGCGCATGCTTTGC-3′	FASMAC	Custom order
Ala-TGC-R: 5′-AACCCGGGGCCTCATACATG-3′	FASMAC	Custom order
Ala-AGC-F: 5′-AGTGGTAGAGCGCGTGCTTA-3′	FASMAC	Custom order
Ala-AGC-R: 5′-CCGGGGCCTCGTGCATGCT-3′	FASMAC	Custom order
Ala-CGC-F: 5′-TGGTAGAGCGCATGCTTCGC-3′	FASMAC	Custom order
Ala-CGC-R: 5′-AACCCGGGACCTCATACATG-3′	FASMAC	Custom order
Thr-AGT-F: 5′-GGTTAAAGCGCCTGTCTAGT-3′	FASMAC	Custom order
Thr-AGT-R: 5′-AACCCAGGATCTCCTGTTTA-3′	FASMAC	Custom order
Thr-CGT-F: 5′-TGGTAAGGCGTCGGTCTCGT-3′	FASMAC	Custom order
Thr-CGT-R: 5′-AACCCGCGATCTTCGGTTTA-3′	FASMAC	Custom order
Gly-TCC-F: 5′-GGTGAGCATAGCTGCCTTCC-3′	FASMAC	Custom order
Gly-TCC-R: 5′-GAACCCGGGTCAACTGCTTG-3′	FASMAC	Custom order
Gly-CCC-F: 5′-TGGTAGAATTCTCGCCTCCC-3′	FASMAC	Custom order
Gly-CCC-R: 5′-GAACCCGGGTCTCCCGCGTG-3′	FASMAC	Custom order
Gly-GCC-F: 5′-TGGTAGAATTCTCGCCTGCC-3′	FASMAC	Custom order
Gly-GCC-R: 5′-GAACCCGGGCCTCCCGCGTG-3′	FASMAC	Custom order
Lys-TTT-F: 5′-GTCGGTAGAGCATCAGACTT-3′	FASMAC	Custom order
Lys-TTT-R: 5′-CCTGGAACCCTCAGATTAAA-3′	FASMAC	Custom order
Lys-CTT-F: 5′-CGGTAGAGCATGAGACTCTT-3′	FASMAC	Custom order
Lys-CTT-R: 5′-AACCCATGACCCTGAGATTA-3′	FASMAC	Custom order
Asn-GTT-F: 5′-GGTTAGCGCGTTCGGCTGTT-3′	FASMAC	Custom order
Asn-GTT-R: 5′-AACCACCAACCTTTCGGTTA-3′	FASMAC	Custom order
Asp-GTC-F: 5′-GGTGAGTATCCCCGCCTGTC-3′	FASMAC	Custom order
Asp-GTC-R: 5′-GAACCCCGGTCTCCCGCGTG-3′	FASMAC	Custom order
Gln-CTG-F: 5′-GGTTAGCACTCTGGACTCTG-3′	FASMAC	Custom order
Gln-CTG-R: 5′-GAACTCGGATCGCTGGATTC-3′	FASMAC	Custom order
Glu-CTC-F: 5′-GGTTAGGATTCGGCGCTCTC-3′	FASMAC	Custom order
Glu-CTC-R: 5′-GAACCCGGGCCGCGGCGGTG-3′	FASMAC	Custom order
Pro-AGG-F: 5′-GGGTATGATTCTCGCTTAGG-3′	FASMAC	Custom order
Pro-AGG-R: 5′-AACCCGGGACCTCTCGCACC-3′	FASMAC	Custom order
Phe-GAA-F: 5′-TGGGAGAGCGTTAGACTGAA-3′	FASMAC	Custom order
Phe-GAA-R: 5′-AACCAGGGACCTTTAGATCT-3′	FASMAC	Custom order
Val-AAC-F: 5′-GGTTATCACGTTCGCCTAAC-3′	FASMAC	Custom order
Val-AAC-R: 5′-AACCGGGGACCTTTCGCGTG-3′	FASMAC	Custom order
Tyr-GTA-F: 5′-TGGTAGAGCGGAGGACTGTA-3′	FASMAC	Custom order
Tyr-GTA-R: 5′-AACCAGCGACCTAAGGATCT-3′	FASMAC	Custom order
Trp-CCA-F: 5′-CGGTAGCGCGTCTGACTCCA-3′	FASMAC	Custom order
Trp-CCA-R: 5′-AACACGCAACCTTCTGATCT-3′	FASMAC	Custom order
Leu-CAG-F: 5′-GTCTAAGGCGCTGCGTTCAG-3′	FASMAC	Custom order
Leu-CAG-R: 5′-CTCCAGGGGAGACTGCGACC-3′	FASMAC	Custom order
Ile-AAT-F: 5′-GGTTAGAGCGTGGTGCTAAT-3′	FASMAC	Custom order
Ile-AAT-R: 5′-AACCCGCGACCTTGGCGTTA-3′	FASMAC	Custom order
Cys-GCA-F: 5′-GGTAGAGCATTTGACTGCAG-3′	FASMAC	Custom order
Cys-GCA-R: 5′-GAACCAGGGACCTCTTGATC-3′	FASMAC	Custom order
Met-CAT-F: 5′-AGGCAGCGCGTCAGTCTCAT-3′	FASMAC	Custom order
Met-CAT-R: 5′-AACTCACGACCTTCAGATTA-3′	FASMAC	Custom order
His-GTG-F: 5′-GGTTAGTACTCTGCGTTGTG-3′	FASMAC	Custom order
His-GTG-R: 5′-GAACCGAGGTTGCTGCGGCC-3′	FASMAC	Custom order
Ser-AGA-F: 5′-GGTTAAGGCGATGGACTAGA-3′	FASMAC	Custom order
Ser-AGA-R: 5′-GGGGAGACCCCAATGGATTT-3′	FASMAC	Custom order
Ser-CGA-F: 5′-GGTTAAGGCGTTGGACTCGA-3′	FASMAC	Custom order
Ser-CGA-R: 5′-GGGGAGACCCCATTGGATTT-3′	FASMAC	Custom order
Arg-TCT-F: 5′-ATGGATAGCGCATTGGACTT-3′	FASMAC	Custom order
Arg-TCT-R: 5′-CCCACAACCTTTGAATTAGA-3′	FASMAC	Custom order
Arg-TCG-F: 5′-ATGGATAAGGCGTCTGACTT-3′	FASMAC	Custom order
Arg-TCG-R: 5′-CCCTCAATCTTCTGATCCGA-3′	FASMAC	Custom order
Arg-CCG-F: 5′-AATGGATAAGGCGTCTGATT-3′	FASMAC	Custom order
Arg-CCG-R: 5′-CCCTCAATCTTCTGATCCGG-3′	FASMAC	Custom order
28S rRNA-F: 5′-CAGGGGAATCCGACTGTTTA-3′	FASMAC	Custom order
28S rRNA-R: 5′-ATGACGAGGCATTTGGCTAC-3′	FASMAC	Custom order

**Recombinant DNA**		

pcDNA-V5-TEV-NEMF	Udagawa et al., 2021	N/A
pcDNA-V5-NEMF	Udagawa et al., 2021	N/A
pcDNA-V5-TEV-NEMF D96A	Udagawa et al., 2021	N/A
p5FBG-Nonstop	Saito et al., 2013	N/A

**Other**		

245 mm (500 cm^2^) Square dish	Corning	431110
Plastic dough scraper	Amazon (nescope)	B08KQG77CP
Open-top polyclear tubes for SW28 rotor	Seton	7052
Gradient Master	BioComp	108
Tube holder, long cap, and marker block for SW28	BioComp	105-925-R
Piston Gradient Fractionator	BioComp	152
Ultracentrifuge	Beckman	XL-A
Magnetic rack	Bio-Rad	1614916
96-Well plate	Sorenson	23080
Nanodrop	Thermo	ND-ONE-W
CFX Connect Real-Time PCR System	Bio-Rad	1855201J1

## Materials and equipment

The stock solutions and buffers below should be prepared in an RNase-free condition. The DEPC-treated water can be used, although it is not essential.

**Table:** Cycloheximide Solution

Reagent	Final concentration	Amount
cycloheximide (Nacalai)	50 mg/mL	250 mg
DMSO	n/a	5 mL
**Total**	**n/a**	**5 mL**

**CRITICAL:** Cycloheximide is toxic and follow institutional guideline for proper disposal. This stock solution should be kept at –20°C for up to a year. Thaw the solution at 20°C–24°C before use.

**Table:** D-PBS

Reagent	Final concentration	Amount
D-PBS powder without Ca and Mg (Nacalai)	n/a	9.6 g
ddH_2_O	n/a	1 L
**Total**	**n/a**	**1 L**

**CRITICAL:** D-PBS should be autoclaved and kept at 4°C for up to a year.

***Alternatives:*** Any other commercially available or homemade D-PBS can be used.

**Table:** Lysis Buffer

Reagent	Final concentration	Amount
HEPES-KOH, pH 7.4 (1 M)	25 mM	1.25 mL
NaCl (5 M)	150 mM	1.5 mL
MgCl_2_ (1M)	5 mM	0.25 mL
Igepal CA-630	1%	0.5 mL
ddH_2_O	n/a	46.5 mL
**Total**	**n/a**	**50 mL**

**CRITICAL:** Lysis buffer can be prepared in advance and stored at 4°C for up to 1 year. When needed, Lysis buffer is supplemented with the protease inhibitor solution, RNasin RNase inhibitor (Promega), and/or the cycloheximide solution immediately before use at the ratio of 10 μL per mL, 3 μL per mL, and 2 μL per mL, respectively.

**Table:** 10% and 35% Sucrose Buffer

Reagent	Final concentration	Amount
Sucrose	10% and 35% (w/v)	5 g and 17.5 g
NaCl (5 M)	150 mM	1.5 mL
MgCl_2_ (1M)	5 mM	0.25 mL
Igepal CA-630	1%	0.5 mL
DTT (1M)	1 mM	50 μL
Cycloheximide (50 mg/mL)	100 μg/mL	100 μL
ddH_2_O	n/a	Adjust up to 50 mL
**Total**	**n/a**	**50 mL**

**CRITICAL:** Sucrose Buffers should not be stored for a long period as it can affect the gradient pattern and the fractionation. Prepare them one day before or on the day of the cell lysis and stored at 4°C until use. DTT and cycloheximide should be added to the buffer immediately before use.

**Table:** Buffer TEV

Reagent	Final concentration	Amount
HEPES-KOH, pH7.5 (1M)	25 mM	0.25 mL
NaCl (5 M)	10 mM	20 μL
MgCl_2_ (1M)	5 mM	50 μL
Glycerol (100%)	5%	0.5 mL
ddH_2_O	n/a	9.18 mL
β-mercaptoethanol (14 M)	7 mM	see below
**Total**	**n/a**	**10 mL**

**CRITICAL:** Buffer TEV without β-mercaptoethanol can be prepared in advance and stored at 4°C for up to a year. β-mercaptoethanol (0.5 μL / mL) should be added immediately before use.

## Step-by-step method details

### Transfection of cells

**Timing: 24 h**

In order to purify the NEMF-60S RNC, V5-TEV-NEMF and control vector (V5-TEV-NEMF D96A or V5-NEMF) are transfected to the cells prepared above. In our case, the plasmid expressing β-globin nonstop protein which is CAT-tailed by NEMF is co-transfected with the vectors expressing NEMF (Udagawa et al., 2021). Two 500 cm^2^ plate cultures are used per sample. This culturing scale usually gives us approximately 100–200 ng of the RNA at the end of the purification which is sufficient for the detection of the tRNAs in the NEMF-60S RNC by RT-qPCR. Depending on the experiment, you may change the choice of tag attached to the protein of your interest. If you use the cells expressing tagged proteins or an antibody against the endogenous protein for immunoprecipitation, you may omit this step.

1. For each 500 cm^2^ plate, prepare the plasmid solution as below.
  a. Add 150 μg of pcDNA-V5-TEV-NEMF wild type, the D96A mutant or the V5-NEMF wild type without a TEV protease recognition motif in a 50 mL tube.
  b. Add 50 μg of p5FBG-Nonstop per tube.
  c. Add 7 mL of Opti-MEM per tube and mix gently by pipetting.
2. For each 500 cm^2^ plate, prepare the PEI-Max solution as below.
  a. Add 600 μL of PEI-Max transfection reagent in a 50 mL tube.
  b. Add 7 mL or Opti-MEM per tube and mix gently by pipetting.
  c. Stand at 20°C–24°C for 5 min.
3. After the incubation of PEI-Max solution for 5 min, mix the plasmid solution and the PEI-Max solution by pipetting and stand at 20°C–24°C for 20 min.
4. Add the transfection complex prepared above to the cell cultures on 500 cm^2^ plates.
5. Replace the medium with 75 mL of DMEM with 10% FBS 4 h after adding the transfection complex, and incubate for 24 h.

***Note:*** All plasmids should be prepared using the transfection-grade plasmid purification kit such as Nucleobond Xtra Midi (Takara) and stored at the concentration of 1 mg/mL at –20°C.

### Preparation of the anti-V5-Protein G Dynabeads

**Timing: 1.5 h**

The anti-V5-Protein G Dynabeads for the immunoprecipitation should be prepared prior to the cell lysis or during sucrose density gradient centrifugation. The protocol below is for two samples (experimental sample and control sample), two 500 cm^2^ cell cultures per sample.

6. Transfer 300 μL of Protein G Dynabeads (75 μL per 500 cm^2^ plate) in a 1.5 mL tube.
7. Place the tube on a magnetic rack, let it stand for a minute, and discard the supernatant by pipetting.
8. Take the tube away from a magnetic rack (BioRad, or its equivalent) and add 600 μL of Lysis buffer and 60 μL of anti-V5 antibody (15 μL per plate).
9. Rotate at 10 rpm at 4°C for 1 h.
10. Place the tube on a magnetic rack, let it stand for a minute, and discard the supernatant by pipetting.
11. Take the tube away from a magnetic rack and wash the beads with 600 μL of Lysis buffer, twice
12. Add 250 μL of Lysis buffer and keep it on ice.

### Preparation of sucrose gradients

**Timing: 15 min**

Prior to the cell lysis, 10%–35% sucrose gradients for the fractionation of 60S ribosomal complexes should be prepared. We use Gradient Master (Biocomp) for making sucrose gradients. This machine allows us to make a variety of density gradients in a convenient and reproducible manner. However, a traditional hand-made method for making gradients can be used alternatively.

13. Prepare one 10%–35% linear sucrose gradient per sample in open-top polyclear tubes (Seton).
  a. Mark a line on the side of the open-top polyclear tubes for SW28 using Marker Block (Biocomp).
  b. Place the tubes on a tube holder (Biocomp).
  c. Pour 10% sucrose until the marked line.
  d. Layer 35% sucrose from the bottom of the tube using a syringe.
  e. Cap the tubes with Long caps for SW28 tubes (Biocomp).
  f. Place the tubes on a tube rack on Gradient Master (Biocomp).
  g. Run a program for 10%–35% linear sucrose gradient.
  h. After the run program, the gradients on a tube holder are placed at 4°C until use.

***Note:*** The gradients can be kept at 4°C for several hours, but we recommend making them just before starting cell lysis.

### Cell lysis

**Timing: 1 h**

Before cell lysis, the cells may be treated with a drug such as cycloheximide in order to ‘freeze’ the ribosome complex. The cell lysis should be done on ice as quickly and carefully as possible to avoid the dissociation of the complexes and the degradation of proteins and RNAs.

14. Decant the medium, pour 30 mL of prewarmed D-PBS supplemented with 100 μg/mL cycloheximide per plate, put the plate back to the 5% CO2 incubator for 10 min.
15. Decant the D-PBS, place the plate on ice, and add 15 mL of ice-cold D-PBS supplemented with 100 μg/mL cycloheximide.
16. Scrape the cells and transfer the cell suspension into a 50 mL tube.
17. Repeat for all plates and collect the cells from same genotype in a same 50 mL tube.
18. Spin down the cells at 5000 *g* for 1 min and aspirate the PBS carefully.
19. Resuspend the cell pellet with 1 mL of Lysis buffer per sample (two 500 cm^2^ plates) supplemented with protease inhibitors, RNasin RNase inhibitor (Promega), and cycloheximide, transfer the lysate into a 2 mL microcentrifuge tube per sample, and keep them on ice for 10 min.
20. Centrifuge at 20000 *g* for 10 min at 4°C and collect the supernatant in a new 2 mL tube.
21. Repeat the centrifugation and collect the supernatant in a new 1.5 mL tube.
22. Keep 20 μL of the lysate as input, add 280 μL of ddH_2_O and 300 μL of acidic phenol (pH 4.2), vortex vigorously, and keep on ice until RNA extraction step below.

***Note:*** When replacing the medium with D-PBS, just decant the medium and directly, but carefully, pour the D-PBS in a 50 mL tube to the plate. Do not use an aspirator and a pipettor, as it takes too much time and the cells can be stressed. When scraping the cells, use a scraper of appropriate size, which significantly shorten the time required for collecting the cells. We usually use a large plastic dough scraper for cooking (see key resources table for an example). The scraper should be kept on ice before use.

### Sucrose density gradient fractionation of the 60S ribosome complexes

**Timing: 4 h**

This step significantly enhances the specificity of the tRNA detection in the NEMF-60S RNC, possibly because NEMF is predominantly localized to the 60S ribosomes (Udagawa et al., 2021). Besides, large amount of free tRNAs which are not bound to the ribosomes can be eliminated by this fractionation, and thus reduces non-specific detection of free tRNAs bound to the immunoprecipitation beads. However, depending on the experiment, you may omit this step and directly proceed to the immunoprecipitation.

23. Layer the lysate from two 500 cm^2^ plates per sample on top of the 10%–35% sucrose gradient.
24. Centrifuge at 27000 *g* for 2.5 h at 4°C with a SW28 rotor with maximum acceleration and deceleration.
25. After the centrifugation, the sucrose gradients are fractionated by Piston Gradient Fractionator (Biocomp) according to the manufacture’s instruction.
26. Pool the 60S ribosome fractions and transfer to a 15 mL tube per sample (about 3 mL per sample).

**CRITICAL:** All lysate (normally approximately 1 mL), after keeping the input samples aside, should be loaded to the gradient. The sample tubes must be balanced before proceeding with centrifugation. If the amount of lysate between the samples varies by more than 100 μL, adjust the balance by carefully removing that amount of sucrose solution from the top of the gradient.

***Note:*** Other gradient fractionators can be used. However, we do not recommend correcting the 60S fractions manually using a syringe to avoid the contamination of other fractions.

**CRITICAL:** We use a UV detector to monitor the ribosomal fractions and correct the 60S ribosomal fractions, the third peak from the top, not to contaminate 40S, the second peak after the first highest peak, and 80S ribosomal fractions, the fourth peak which often overlaps with 60S ribosomal fractions. Since the 60S and 80S ribosomal fractions overlap, we do not include the boundary fraction between the third and fourth peaks.

### Immunoprecipitation of the NEMF-60S RNC

**Timing: 3 h**

The NEMF-60S RNC is purified by the immunoprecipitation of the V5-TEV-NEMF using anti-V5 antibody from the 60S ribosome fraction prepared above. The immunoprecipitated complexes are eluted by digesting V5-TEV-NEMF with AcTEV protease (Thermo) or the equivalent (Matsuo et al., 2017; Udagawa et al., 2021). When this protocol is used for other purpose, you may use other tag or an antibody against endogenous protein of your interest. However, it is important that you elute the complexes from the beads at the end of this step, in order to detect the bound tRNAs specifically in the complexes. RNA extraction directly from the beads will increase non-specific detections of tRNAs.

27. Add 125 μL of the anti-V5-Protein G Dynabeads prepared above to the 60S fraction in a 15 mL tube per sample
28. Rotate the tubes at 10 rpm for 1 h at 4°C.
29. Place the tubes on a magnetic rack, let it stand for a minute, and discard the supernatant carefully by pipetting.
30. Take the tubes away from a magnetic rack, wash the beads with 700 μL of Lysis buffer supplemented with RNasin RNase inhibitor per sample by gentle pipetting, and transfer the beads into a new 1.5 mL tube per sample.
31. Similarly, wash the beads with 700 μL of Lysis buffer supplemented with RNasin RNase inhibitor three more times.
32. Wash the beads with 700 μL of Buffer TEV once.
33. Elute the NEMF-60S RNC by digesting V5-TEV-NEMF with 70 units of AcTEV protease (Thermo) or the equivalent in 300 μL of Buffer TEV per sample.
34. Rotate the tubes at 10 rpm for 2 h at 4°C.
35. Place the tube on a magnetic rack and collect the eluate in a new 1.5 mL tube per sample.

***Note:*** At the end of this step, you have the NEMF-60S RNC ready for RNA/protein analysis. For the first time of the experiments, you may confirm the purity of the complex by western blotting (Figure 1A). When performing western blotting, the eluted proteins may be concentrated by TCA precipitation.

**CRITICAL:** To avoid non-specific binding, the incubation period of the beads with the 60S fraction should not be longer than 1 h.

**Pause point:** The eluted complexes can be stored at –20°C only after the addition of an equal volume of acidic phenol (pH 4.2) for RNA analysis and ice-cold 20% TCA for protein analysis.

### RNA extraction

**Timing: 2 h**

In order to detect the tRNAs in the NEMF-60S RNC, RNA extraction is carried out by acidic phenol method. You may also use commercially available RNA purification kit such as TRIzol (Thermo) with Direct-zol RNA miniprep kit (Zymo research). Input RNA prepared at step 9 of ‘Cell lysis’ above should be processed together with the eluted samples.

36. Add equal volume (300 μL) of acidic phenol (pH 4.2) to the eluted complexes above, vortex rigorously, centrifuge at 15000 *g* for 10 min at 4°C together with the input sample.
37. Collect approximately 280 μL of the aqueous phase, add 300 μL of chloroform, vortex rigorously, centrifuge at 15000 *g* for 10 min at 4°C.
38. Collect approximately 250 μL of the aqueous phase, add 0.5 μL of 20 mg/mL glycogen, 30 μL of 3 M sodium acetate, pH 5.5, and 750 μL of ethanol in order, mix well after each addition, vortex rigorously after adding ethanol, keep the tubes at –80°C for 1 h.
39. Centrifuge at 15000 *g* for 10 min at 4°C, carefully aspirate the supernatant, add 1 mL of 75% ethanol to the pellet without vortexing, and centrifuge again at 15000 *g* for 10 min.
40. Air dry and resuspend the pellet in 20 μL of ddH_2_O for the eluted sample and 200 μL for the input sample.
41. Quantify the RNAs by Nanodrop.

***Note:*** Nuclease-free water, for example one from Ambion, instead of ddH2O might be used from this step and after.

***Note:*** With this scale of the purification, we usually obtain 200 μL of the input RNAs at the concentration of 200–250 ng/μL and 20 μL of the eluted RNAs at the concentration of 5–10 ng/μL. The concentration of the eluted RNA is the nearly lower limit of Nanodrop quantification, but is sufficient for tRNA detection by RT-qPCR below. The quantification can be performed more accurately by fluorescent-based methods or Bioanalyzer (Agilent), if needed.

**Pause point:** The RNA samples can be stored at –80°C after the addition of 100% ethanol (step 3) or 75% ethanol (step 4). The resuspended RNAs can also be stored at –80°C for weeks.

### RT-qPCR for tRNAs

**Timing: 4 h**

Finally, measure the level of each tRNA in the NEMF-60S RNC by RT-qPCR (Figure 1B). The protocol described below uses conventional RT-qPCR enzymes with custom primers for each human tRNA and we were able to detect most tRNA species by this protocol. tRNA sequences were obtained from GtRNAdb (Chan and Lowe, 2016). All primer pairs are designed to amplify the anticodon stem-loop, with the 3′ end of both forward and reverse primers positioned within the anticodon loop. We chose this region because of the relatively high sequence variation among tRNA species. However, note that we could not obtain specific amplification for some tRNA species, including tRNA_i_^Met^, although we have not tested all possible primer sequences. With the RNAs prepared above, you can examine up to 40 different tRNA species.

42. Dilute and denature the input and the eluted RNA samples.
  a. Dilute 20 μL of the eluted RNA and 2 μL of the input RNA with ddH_2_O up to 100 μL.
  b. Heat at 75°C for 10 min and immediately chill on ice

***Note:*** Although the RNAs are denatured prior to the reverse transcription reactions below, this additional denaturing step often enhances the qPCR amplification of tRNAs, probably because it helps denature tRNA secondary structure and improves accessibility of the reverse transcriptase.

43. Perform reverse transcription reactions as below.
  a. Mix 2.5 μL of the diluted RNA with 2 μL of 10 μM reverse primer for each tRNA in a PCR tube, keep at 65°C for 5 min in a PCR machine and immediately chill on ice.
  b. Prepare the reaction mixtures containing 3 μL of 5× ReverTra Ace RT buffer (TOYOBO), 3 μL of 10 mM dNTPs, 0.375 μL of RNasin RNase inhibitor, 0.375 μL of ReverTra Ace Reverse Transcriptase (TOYOBO), 3.75 μL of ddH_2_O per sample for the number of samples.
  c. Add 10.5 μL of the reaction mixture to the denatured RNA/primer mixture and start the reaction at 55°C for 30 min, 95°C for 5 min, and 4°C forever on a PCR machine.

**Pause point:** Although not recommended, the cDNAs prepared above can be stored at 4°C for a week. Longer storage can affect the qPCR result.

**Figure 2:**
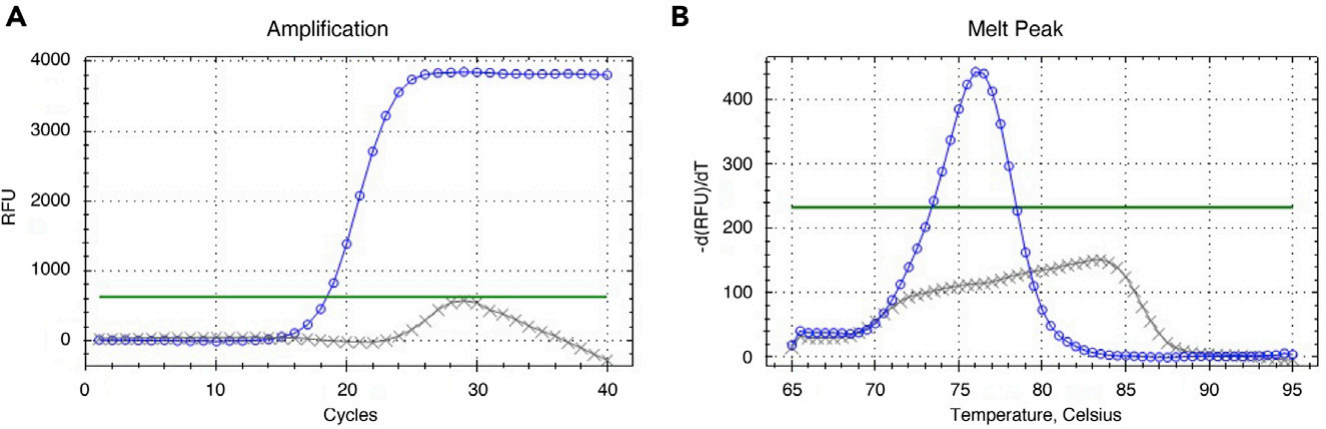
Typical amplification and melting curve analysis of normal and failed qPCR reactions (A) The amplification curves of normal (tRNA^Gly^_TCC_, blue line with circle symbols) and failed (Med-tRNAi^Met^, gray line with cross symbols) reactions. Green line indicates the threshold line for quantification which is automatically displayed by the qPCR system (BioRad). (B) Melting curve analysis of normal (tRNA^Gly^_TCC_, blue line with circle symbols) and failed (tRNAi^Met^, gray line with cross symbols) reactions. Green line indicates the threshold line for quantification.

44. Perform qPCR reactions as below using KAPA SYBR Fast qPCR kit (Nippon Genetics) and BioRad CFX Connect system (BioRad) or the equivalent.
  a. Prepare the reaction mixtures for each tRNA containing 7.5 μL of 2× KAPA SYBR Fast qPCR mixture, 0.3 μL of 10 μM forward and reverse primers, and 4.9 μL of ddH_2_O per sample for the number of samples.
  b. Add 2 μL of the cDNA for the number of tRNAs to be tested in a 96 well qPCR plate.
  c. Mix 13 μL of the reaction mixture with the cDNA in the 96 well plate, place the plate in a qPCR machine (BioRad), and start the preinstalled 2 step protocol with a melt curve step below (Figure 2).

**Table:** 

qPCR cycling conditions			
Cycling steps	Temperature	Time	Cycles
Initial denaturation and enzyme activation	95°C	4 min	1
Denaturing	95°C	10 s	40
Annealing and extension	55°C	30 s	
Denaturing	95°C	10 s	1
Melt curve	55°C–90°C (0.5°C increments)	5 s	1

***Note:*** The levels of individual tRNAs in the experimental sample are compared to those of the control sample based on the Cq values obtained by RT-qPCR. The values may be normalized to an internal control such as 28S rRNA.

***Note:*** We recommend to perform qPCR reactions with technical replicates for each primer sets. At least three biological replicates are needed for proper quantification.

## Expected outcomes

Yeast homolog of NEMF, RQC2, mediates CAT-tailing of the nascent peptides which promotes efficient degradation of the nascent peptides during ribosome-associated quality control (RQC) (Shen et al., 2015). However, it has not been certain whether CAT-tailing can occur in mammalian cells. In our recent article, we demonstrated using the protocol described here that tRNA^Ala^, tRNA^Thr^ , tRNA^Gly^ and several other tRNAs are enriched in the NEMF-60S RNC in HEK293T cells, which suggested that CAT-tailing is conserved in mammalian cells, and that mammalian CAT-tailing utilizes not only alanine and threonine as in budding yeast but also several other amino acids (Udagawa et al., 2021). The most critical step that enhanced specific detection of tRNAs in the NEMF-60S RNC complex was the fractionation of 60S ribosome by sucrose density gradient centrifugation (Figure 1C). It was also important to have an appropriate control sample because you can evaluate only relative levels of individual tRNAs in the experimental sample to the control sample by this method using RT-qPCR. The data shown here uses V5-NEMF without a TEV protease motif which cannot be eluted from the beads as a negative control and the IP enrichment of alanyl tRNAs in the V5-TEV-NEMF sample relative to the V5-NEMF sample was evident (Figure 1B). However, much higher enrichment was observed when V5-TEV-NEMF D96A mutant which lacks CAT-tailing activity was used as a negative control (Figure 1D) (Udagawa et al., 2021). Thus, the protocol described here allows us to identify tRNAs in the ribosomal complexes, but when this protocol is applied to other ribosomal complexes or tRNA-containing complexes, the experimental condition should be carefully considered depending on the purpose of the experiments.

Quantification of tRNAs by RT-qPCR has been thought to be difficult due to the presence of base modifications and strong secondary structure in the tRNA molecules. However, after careful consideration of the primer pairs used for RT-qPCR reactions, we were able to set up the reactions that specifically detect individual tRNAs in a reproducible manner. Important points when designing the specific primers for tRNAs are the size of the amplicon and the location of the tRNA to which the primers hybridize. The amplicon size should be kept as short as possible. The forward and the reverse primers can be overlapped for up to one base to minimize the amplicon size. The primers should be designed to amplify anticodon stem and loop. Using this protocol, relative levels of individual tRNAs can be conveniently measured as shown above. This protocol can also be used to evaluate individual tRNA levels among tissues and cell types (Figure 1E) and thus could be useful for researchers.

## Limitations

The protocol described here aims to identify the tRNA species enriched in the ribosomal complexes. RT-qPCR measurement of the tRNAs is a convenient method to compare the level of each tRNA in cells and in the tRNA-containing complexes. However, since tRNAs are amplified by PCR reactions using specific primer pairs for each tRNA, the information obtained by this method is limited to relative level of each tRNA in the experimental sample compared to the control sample. If you need to know absolute levels of different tRNA species, more unbiased approaches such as tRNA-seq must be employed. It should also be noted again that for some tRNAs, including tRNA_i_^Met^, we could not obtain specific amplification by qPCR, although we have not tested all possible primer sequences. Besides, this method cannot quantitatively distinguish between amino-acid charged and uncharged and base-modified and unmodified tRNAs.

## Troubleshooting

### Problem 1

Low transfection efficiency (Transfection of cells).

### Potential solution

You might increase the amount of DNA and PEI-Max used for transfections up to 1.5 times. However, higher concentration of PEI-Max leads to more toxicity to the cells. You might also use other transfection reagents such as Lipofectamine 3000 (Thermo) and Xfect transfection reagent (Clontech).

### Problem 2

Ribosomal subunits on sucrose gradient are not separated well. (Sucrose density gradient fractionation of the 60S ribosome complexes)

### Potential solution

Keep the amount of the lysate loaded onto the gradient less than 1.2 mL per tube. Usually with the method described above, you get about 1 mL of the lysate and you can apply all the lysate onto one gradient (35.2 mL) per sample. If you get more than 1.2 mL of the lysate, run two gradients per sample.

### Problem 3

Polysomes are not detected (Sucrose density gradient fractionation of the 60S ribosome complexes).

### Potential solution

If you need polysome fractions for immunoprecipitation, keep the cell confluency less than 70%. Over-confluency results in translation repression, thus low level of polysomes on the gradient. You might also use 10%–50% sucrose gradient instead of 10%–35% as described above. Transfection also affects the polysome pattern. After transfecting cells, the level of polysomes relative to monosomes often decreases compared to that in non-transfected cells. If that is the case, you might transduce the cells using lentivirus or adeno-associated virus.

### Problem 4

The amount of RNA purified after immunoprecipitation is low. (Immunoprecipitation of the NEMF-60S RNC)

### Potential solution

This can be caused by various reasons. You might increase the culturing scale, which gives you more lysate and more 60S fractions for immunoprecipitation. You might also increase the antibody and the dynabeads for immunoprecipitation. However, do not extend the incubation period of immunoprecipitation as it can result in increased non-specific binding.

### Problem 5

RT-qPCR assays are inefficient and/or abnormal. (RT-qPCR for tRNAs)

### Potential solution

With the method described above, the Cq values obtained by qPCR for individual tRNAs are usually less than 35 and the amplification curve should be normal (Figure 2). Melting curve analysis of normal reactions should show a single distinct peak, while that of abnormal reactions shows broad and/or multiple peaks. If the Cq is higher than 35, you might observe abnormal curves. In those cases, increase the amount of RNA used for RT reactions and the cDNA for qPCR reactions. When you use new primer pairs, it is recommended that you test them using total RNA for a normal amplification before starting experiments.

## Resource availability

### Lead contact

Further information and requests for resources and reagents should be directed to and will be fulfilled by the lead contact, Toshifumi Inada (toshiinada@ims.u-tokyo.ac.jp).

### Materials availability

This study did not generate new unique reagents.

### Data and code availability

The study did not generate any unique datasets or code.
